# The Emerging Role of Complement Proteins as a Target for Therapy of IgA Nephropathy

**DOI:** 10.3389/fimmu.2019.00504

**Published:** 2019-03-19

**Authors:** Dana V. Rizk, Nicolas Maillard, Bruce A. Julian, Barbora Knoppova, Todd J. Green, Jan Novak, Robert J. Wyatt

**Affiliations:** ^1^Department of Medicine, University of Alabama at Birmingham, Birmingham, AL, United States; ^2^Department of Nephrology, Dialysis, Transplantation, CHU de Saint-Etienne, GIMAP, EA3064, Université Jean Monnet, COMUE Université de Lyon, Rhône-Alpes, France; ^3^Department of Microbiology, University of Alabama at Birmingham, Birmingham, AL, United States; ^4^Department of Immunology, Faculty of Medicine and Dentistry, Palacky University and University Hospital, Olomouc, Czechia; ^5^Department of Pediatrics, University of Tennessee Health Sciences Center, Memphis, TN, United States

**Keywords:** complement, IgA nephropathy, alternative complement pathway, mannan binding lectin complement pathway, IgAN pathogenesis, IgAN, IgAN treatment

## Abstract

IgA nephropathy (IgAN) is the most common form of primary glomerulonephritis worldwide and a common cause of end-stage renal disease. Evaluation of a kidney biopsy is necessary for diagnosis, with routine immunofluorescence microscopy revealing dominant or co-dominant IgA immunodeposits usually with complement C3 and sometimes IgG and/or IgM. IgA nephropathy reduces life expectancy by more than 10 years and leads to kidney failure in 20–40% of patients within 20 years of diagnosis. There is accumulating clinical, genetic, and biochemical evidence that complement plays an important role in the pathogenesis of IgA nephropathy. The presence of C3 differentiates the diagnosis of IgA nephropathy from the subclinical deposition of glomerular IgA. Markers for the activation of the alternative and mannan-binding lectin (MBL) pathways in renal-biopsy specimens are associated with disease activity and portend a worse renal outcome. Complement proteins in the circulation have also been evaluated in IgA nephropathy and found to be of prognostic value. Recently, genetic studies have identified IgA nephropathy-associated loci. Within these loci are genes encoding products involved in complement regulation and interaction with immune complexes. Put together, these data identify the complement cascade as a rational treatment target for this chronic kidney disease. Recent case reports on the successful use of humanized anti-C5 monoclonal antibody eculizumab are consistent with this hypothesis, but a better understanding of the role of complement in IgA nephropathy is needed to guide future therapeutic interventions.

## Introduction

IgA nephropathy (IgAN), initially described by Berger and Hinglais in 1968 (1), is the most common primary glomerulopathy in many countries. IgAN causes end-stage renal disease in 20–40% of the patients within 20 years after diagnosis (2), and reduces life expectancy by 10 years (3). The diagnosis is based on immunofluorescence- or immunohistochemical-microscopic examination showing IgA as the dominant or co-dominant immunoglobulin in the glomerular immunodeposits (4). These deposits may also contain IgG, IgM, or both (4). The IgA is exclusively of the IgA1 subclass (5). Complement component C3 is present in the same distribution as IgA in up to 90% of biopsies (4).

**Figure 1:**
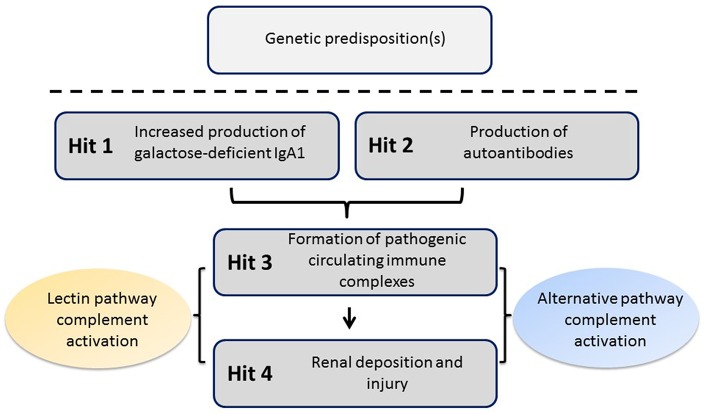
Proposed four-hit pathogenesis of IgAN. Circulatory galactose-deficient IgA1 (Gd-IgA1) (Hit 1) is recognized by specific autoantibodies (Hit 2) to form circulating immune complexes (Hit 3). Some of these immune complexes deposit in the kidneys, thereby leading to mesangial activation, enhanced proliferation of mesangial cells, and ultimately kidney injury (Hit 4). Certain genetic loci have been associated with increased risk for developing IgAN. The activation of the alternative and, at least in some patients, mannan-binding lectin (MBL) pathways by immune complexes is involved in disease pathogenesis.

Recent studies have confirmed an autoimmune nature of IgAN. The most widely accepted mechanism for the pathophysiology of the disease entails four “hits” (Figure 1) (6). The first hit refers to increased levels of circulatory polymeric IgA1 with aberrant *O*-glycosylation of its hinge region. These molecules lack galactose in some *O*-glycans in the hinge region (galactose-deficient IgA1, Gd-IgA1), thus exposing *N*-acetylgalactosamine (GalNAc) as terminal glycan. The second hit is the formation of glycan-specific IgG or IgA1 autoantibodies targeting terminal GalNAc-containing hinge region of Gd-IgA1 (7). The third hit is formation of circulating immune complexes comprising Gd-IgA1 and IgG autoantibody. Other proteins can bind Gd-IgA1, such as the soluble Fcα receptor (sCD89), to form complexes, although it is not clear whether such complexes would activate complement (8). Some of the circulating immune complexes pass through fenestrae in the glomerular capillaries to enter the mesangium where they may incite cellular proliferation of mesangial cells and overproduction of extracellular matrix, cytokines, and chemokines (hit four) that potentially lead to chronic kidney damage. This proposed multi-step process is consistent with the finding that glomerular IgA immunodeposits of patients with IgAN are enriched for Gd-IgA1 (9, 10) and IgG co-deposits are of IgG1 and IgG3 subclasses, as are the circulatory IgG autoantibodies specific for Gd-IgA1 (7, 11).

Key observations in kidney transplantation support the notion that kidneys in IgAN are damaged as innocent bystanders: IgAN frequently recurs in allografts, whereas IgA deposits clear from kidneys of donors with subclinical IgAN within a few weeks after implantation into non-IgAN recipients (12).

IgAN is broadly categorized as primary or secondary, i.e., associated with a systemic disease, be it an infectious, inflammatory, or autoimmune process (13). Within primary IgAN the spectrum of disease varies substantially. The clinical presentation differs between children and adults, and the disease severity as well as gender distribution across ethnic and racial backgrounds differ widely. IgAN can manifest without extra-renal involvement, or as part of a systemic vasculitis phenotype currently referred to as IgA vasculitis with nephritis (previously Henoch Schönlein pupura nephritis) (14). About 5–8% of patients have a first- or second-degree relative with biopsy-proven IgAN or urinary abnormalities suggesting a familial occurrence or genetic predisposition for the disease (15). All these observations raise the possibility that the renal pathology phenotype we call IgAN results from different pathophysiologic processes.

In recent years, mounting pathologic, biochemical, experimental, and genetic findings have supported a pivotal role of complement activation in disease onset and progression of IgAN. In particular, the alternative and mannan-binding lectin (MBL) pathways seem to be involved. These observations, in turn, have generated tremendous interest in targeting complement pathways as an approach to treatment.

## Brief Overview of the Complement-Activation Pathways

Early knowledge of complement proteins stemmed from the 19th-century discovery of a heat-labile component of normal plasma that augmented the opsonization of bacteria by antibodies and enabled antibodies to kill some bacteria (16, 17). The name “complement” was derived from the description of the activity that “complemented” the antibacterial activity of antibodies. The complement system is an important link between innate and adaptive immunity as it participates in immunosurveillance and tissue homeostasis. The system consists of the activation cascade of ~50 proteins located in plasma, tissues, and cells (18–20). Many of these complement-associated proteins have been described as proteases that are activated by proteolytic cleavage. This cascade of proteolytic events must be well-controlled for the system to work properly. A malfunction may result in immunodeficiency or autoimmune manifestations (21–23).

**Figure 2:**
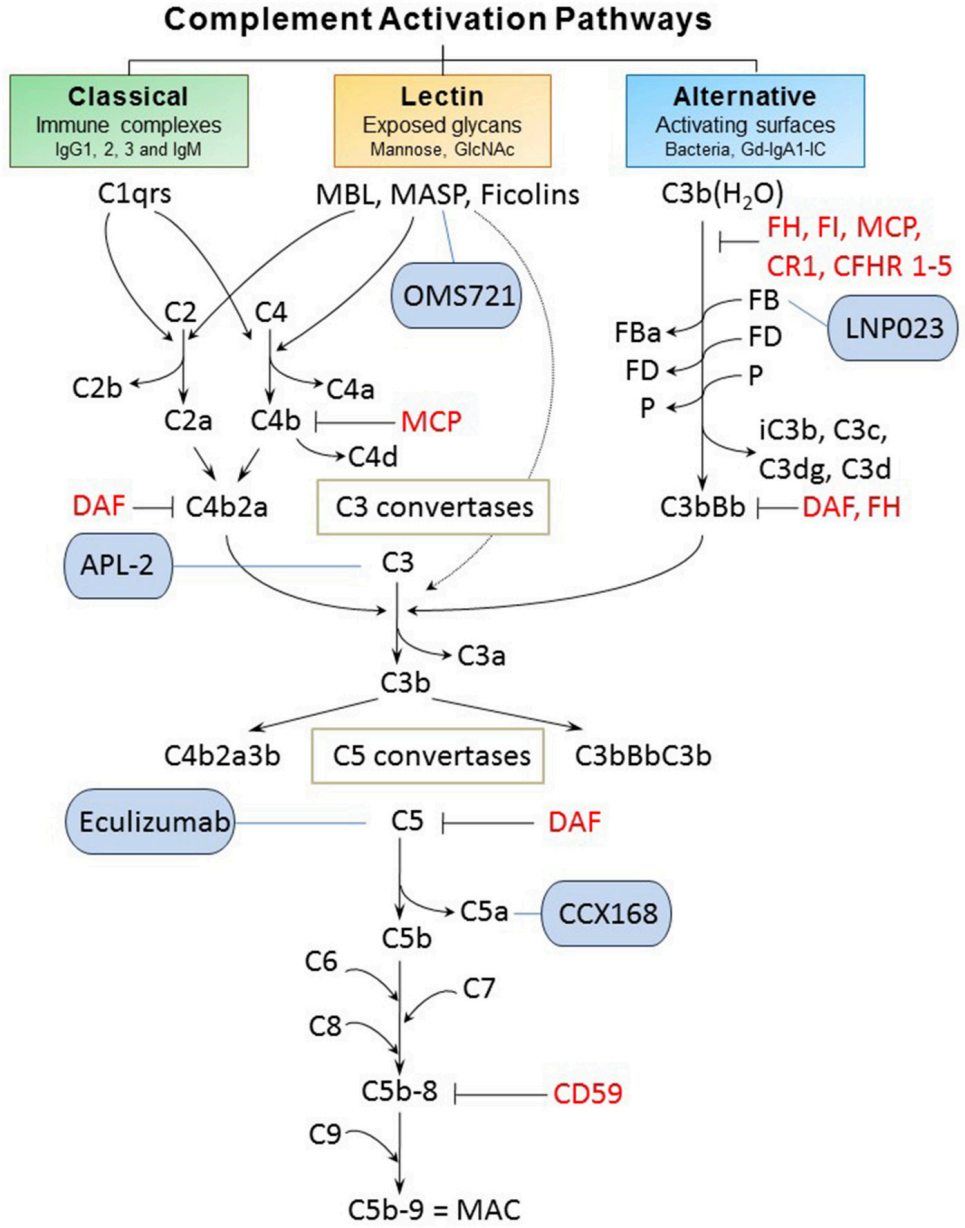
Complement activation pathways and examples of complement-targeting therapeutics for IgAN. The three pathways of complement activation, classical, lectin, and alternative, are initiated by interactions of complement proteins with distinct structures. Complexes of antigen and antibody can activate the classical pathway. Mannan-binding lectin recognizes carbohydrate structures and, upon association with serine proteases (MASP, mannose-associated serine proteases), can activate the lectin pathway. Complement C3 that is covalently bound to microorganism surfaces as C3b initiates the cascade of the alternative pathway. Each pathway can ultimately generate an active C3 convertase, resulting in cleavage of C3 component into C3a and C3b fragments. C3b can interact with C4b2b or C3bBb to produce C5 convertase that cleaves C5 into C5a and C5b fragments. C5b binds to the cell membrane and serves as a platform for assembly of the membrane attack complex (MAC) (24, 25). The formation of MAC can be inhibited by membrane-bound CD59 that binds to C8 and/or C9. Several other regulatory proteins of the complement-activation pathways are shown in red. Five complement-targeting therapeutics are shown in blue rounded rectangles. These reagents include two monoclonal antibodies (eculizumab that blocks cleavage of C5; OMS721 targeting MASP-2 that inhibits its protease activity), a C5a receptor antagonist (CCX168), a low-molecular-weight inhibitor of factor B (LNP023), and an inhibitor of C3 activation (APL-2). CR1, complement receptor 1; CFHR 1-5, complement factor H-related proteins 1-5; DAF, decay-accelerating factor; FB, factor B; FD, factor D; FI, factor I; Gd-IgA1, galactose-deficient IgA1; Gd-IgA1-IC, galactose-deficient IgA1-containing immune complexes; MCP, membrane cofactor protein; P, properdin.

Classical, alternative, and lectin pathways are the three known ways of complement system activation (Figure 2). Each pathway has a different triggering mechanism; however, after creating the C3-activating enzymes (C3 convertases), the pathways share the same sequence of events that culminates with assembly of the membrane-attack complex (MAC) (26). The first activated component of the classical pathway is C1q protein that recognizes an antigen-antibody (IgG or IgM) complex and subsequently binds its partners C1r and C1s to create a protein complex named C1 (C1q:C1r_2_:C1s_2_) (27, 28). C1 has the ability to cleave complement components C2 and C4, into C2a and C4b, respectively, that interact to form C3 convertase (C4b2a). Subsequently, the cleavage of component C3 by participation of C3 convertase produces two proteins. The smaller submit C3a is an anaphylatoxin that mediates inflammation. The larger subunit C3b is an opsonin that binds covalently through a reactive thioester bond to adjacent pathogen molecule and thereby targets it for destruction by phagocytes equipped with receptors for C3b. As the next step of the cascade, C5 convertase is formed by association of C3b with C4b2a or with C3bBb (the product of cleavage from the alternative pathway). C5 convertase then releases the C5a subunit from C5 protein and the remaining C5b fragment initiates formation of the MAC. After the addition of components C6, C7, and C8, the complex C5b-8 is incorporated into the cell membrane, followed by addition of 10–16 units of C9 component that are arranged in the shape of ring, creating a pore in the membrane and leading to cell lysis and death. C3a and C5a cleavage products have inflammatory and chemo-attractant activities exerted through the corresponding C3a and C5a receptors. Moreover, complement functions include facilitation of uptake and destruction of pathogens by phagocytic cells through the specific recognition by complement receptors on phagocytes. There are six types of complement receptors: CR1-4, and C3a and C5a receptors (29, 30).

The alternative pathway is continuously activated at a low level, and is amplified on activating surfaces or in the fluid phase by bacteria, dying cells, and immune complexes. The spontaneous hydrolysis of a thioester bond in C3 component produces C3b(H_2_O) that is tightly controlled by plasma regulatory proteins (factors H [FH] and I [FI]; membrane cofactor protein [MCP, CD46]; complement receptor 1 [CR1, CD35]; decay accelerating factor [DAF, CD55]; and complement factor H-related proteins [CFHRs]) (31). Together, these regulators prevent the formation or enhance the dissociation of the alternative pathway C3 convertase (FH, CR1, and DAF) or serve as cofactors for FI-mediated inactivation of C3b to iC3b (FH, MCP, and CR1). CFHRs compete with FH. Properdin is a positive regulator of the alternative pathway, with its main role of stabilizing the alternative pathway C3 convertase (and C5 convertase). Active convertase C3bBb acts similarly to C4b2a, as it activates C5 convertase that leads to formation of MAC (29, 30).

The lectin pathway is stimulated upon binding of MBL with MBL-associated serine proteases (MASP) or ficolins to specific carbohydrate patterns. MASP-2 can also directly cleave C3 while bypassing the usual sequences to activate C4 and/or C2 (32).

Recent data have shown that renin, an aspartate protease produced by juxtaglomerular apparatus in the kidneys, can also function as a C3 convertase to activate the terminal portion of the complement cascade (33).

## Role of Complement Proteins in IgAN

Complement proteins are activated in IgAN. Immuno-histochemical findings of C3, properdin, C4d, MBL and C5b-9 deposits in mesangium of IgAN biopsy samples, coupled with the general absence of C1q, confirm activation of alternative and lectin pathways rather than classical pathway (24, 34, 35).

The complement cascade is regulated at several levels to prevent unwanted (uncontrolled) activation. As noted above, one of the controlling steps of the alternative pathway relies on complement FH that is present in plasma and on tissue surfaces. It has two functions, stabilizing complexes with C3b and accelerating dissociation of C3bBb. CFHRs are sequentially similar to FH and can compete with FH for C3b binding. These proteins have been studied as possible risk factors of IgAN. In a study with 1,126 IgAN patients, higher circulating levels of CFHR-5 were associated with IgAN development and progression (36, 37). Another study showed that plasma levels of FH antagonists FHR-1 and the FHR-1/FH ratio were elevated in patients with IgAN and associated with disease progression, whereas the plasma level of FHR-5 and the FHR-5/FH ratio were not. However, elevated levels of FHR-5 correlated with poor response to immunosuppressive therapy (38, 39). Moreover, gene deletions of *CFHR1,3* (protective alleles) and some rare variants of CFHR5 are associated with IgAN susceptibility.

### Complement Activation by IgA, IgG, IgM, and Immune Complexes

Human IgG antibodies can have pro- and anti-inflammatory activities, depending on the engagement of Fcγ receptors and the activation of the complement system, which, in turn, depends on the IgG subclass, hexamerization, glycosylation, and antigen density (40–49). Activation of the classical pathway by IgG (and IgM) isotypes (mostly driven by IgM and IgG1 and IgG3 subclasses, and hexameric IgG) results in production of pro-inflammatory C3a and C5a (50). This process then triggers recruitment of effector cells wherein the deposition of C3b on target cells enables recognition by C3b receptors on phagocytic and antigen-presenting cells. Moreover, the capacity of IgG to activate complement further depends on glycosylation of its Fc segment. For example, IgG can also activate the lectin pathway if the Fc glycans consist of complex *N*-glycans with terminal *N*-acetylglucosamine (i.e., galactose-deficient *N*-glycans) (51).

In contrast to IgG and IgM, human IgA does not activate complement in the fluid phase and is considered anti-inflammatory. However, differential IgA glycosylation of monomeric and polymeric IgA bound in immune complexes may positively or negatively impact complement activation (52). Mouse models have shown that autoantibodies can activate the alternative pathway and induce cell lysis and tissue damage or target autologous complement components. Such autoantibodies may play a role in several diseases, especially vascular diseases (53). A passive mouse model of IgAN that uses pre-formed immune complexes comprised of human Gd-IgA1 and human IgG autoantibody specific for Gd-IgA1 induces hematuria and proteinuria; moreover, the glomeruli exhibit mesangial hypercellularity and deposits of IgG, IgA1, and C3 (14).

Activation of the complement system by immune complexes is less well-understood. The relative representation of immunoglobulin isotypes in an immune complex may co-determine which complement pathway or pathways are activated (54). This finding may arm researchers with tools and approaches for use in complement modulation with therapeutics (55). Selected examples relevant for IgAN are shown in Figure 2.

## Complement Proteins and Complement Fragments in Pathology

### Presence of Complement Elements in Glomeruli

A characteristic immunofluorescence-microscopy feature of IgAN renal biopsies is the almost universal glomerular deposition of some complement proteins with IgA. C3 is the most abundant, found in up to 90% of cases (56–60). C3 co-deposits could also be considered a biomarker of actual IgAN in comparison to isolated IgA deposition without renal injury. Interestingly, a European necropsy study from 753 deaths due to suicide or violent deaths (excluding persons with secondary forms of IgAN) found asymptomatic IgA deposition in the kidneys of 6.9% of individuals (61). Of those, only 4 were C3-positive (0.5%). A study from Japan evaluated the prevalence of IgA deposition among 510 kidney donors whose renal allografts were biopsied at the time of implantation. The frequency of subclinical IgA deposition was 16.1%, with concomitant C3 deposition reported in 16 individuals (3%) (62). These studies show that complement activation may distinguish isolated from nephritogenic IgA deposition. Notably, the intensity of C3 deposition by immunofluorescence studies can be influenced by genetic variations in the complement-encoding genes. Patients with at least one allele for a large deletion in the genes encoding CFHR proteins (Δ*CFHR3,1*) have less glomerular immunofluorescence staining for C3 compared to individuals with two wild-type alleles (63).

Apart from C3, other complement elements can be co-deposited with IgA. The presence of FH or properdin, suggesting activation of the alternative pathway, has been frequently found in mesangial areas of IgAN patients (59, 64, 65). More recently, mass-spectrometric analysis of micro-dissected glomeruli from IgAN kidney-biopsy specimens showed significant amounts of C3 and C5 as well as all of the complement elements located downstream from the activation cascade (C6 to C9) when compared to biopsies of normal kidneys (66). This result confirms the presence of C5b-9 in IgAN glomeruli, as shown in early immunostaining-based studies (64, 67, 68). The most important point from this study is the accumulation of alternative-pathway regulation proteins, such as FH, and also CFHR 1,2,3, and 5. Moreover, using targeted proteomic profiling, a reduced abundance of complement receptor 1 (CR1) was detected in biopsy specimens from IgAN patients with progressive vs. non-progressive disease (66, 69).

The lectin pathway is activated in some patients with IgAN (70). This subset of patients exhibits mesangial deposition of C4d, MBL, MBL-associated serine proteases (MASPs) 1 and 2 and L-ficolin (70). In a large multi-center Spanish cohort of 283 IgAN patients, C4d deposition was found in 38% of cases (71).

Hallmarks of classical pathway activation, such as C1q, are usually not detected in the glomeruli of patients with IgAN (57), although it may be found in biopsies with advanced glomerulosclerosis (72). Therefore, the presence of glomerular C4d in IgAN suggests activation of the lectin pathway rather than the classical pathway.

### Association of Complement Protein Deposition With Disease Severity and Prognosis

The intensity of mesangial C3 deposition has been negatively correlated with renal survival in a retrospective study of 343 Korean IgAN patients (58). A trend toward a similar finding was reported in a large study of French patients, although the finding did not reach statistical significance (63). The prognostic implications of detection of early (C3b, C3c, iC3b) vs. late (C3d) proteolytic products in mesangial deposits remain matters of debate. One study reported more active disease associated with C3c deposition compared to C3d (73). Another recent study found C3d and C3c/C3b/iC3b were independently associated with progressive disease in a small number of patients (38). C5b-9 deposits have been associated with disease activity in some immunostaining-based studies (38, 74). A recent study using a proteomic approach also confirmed that the presence of glomerular complement protein deposition was associated with progressive IgAN (66).

The deposition of regulatory proteins of the alternative pathway has also been related to the activity of IgAN. Patients with progressive disease had more FH and CFHRs 2 and 5 and less CR1 as assessed by mass spectrometry (66). An immunostaining approach very recently confirmed deposition of CFHR1 and CFHR5, with frequency of the finding dependent on disease severity (38). Notably, glomerular deposition of CFHR5 was significantly more frequent in biopsies from 19 patients with progressive IgAN compared to 18 stable counterparts (odds ratio [OR] 13.4 [2.2–66.9]). On the contrary, FH was less frequently deposited (OR 0.1 [0.08–0.87]) with progressive disease. These findings suggest an imbalance between CFHR5 and FH that may accentuate disease severity. Indeed, CFHRs are sequentially similar to FH and can compete with FH for C3b binding, but lack some regulatory functions. For example, CFHR1, compared to FH, lacks FI cofactor activity and the capacity to accelerate decay of C3 convertase. CFHR5 can act as FI cofactor but only at supra-physiologic concentrations, thus being less efficient than FH. Nevertheless, the promising results of these early studies need to be confirmed in larger cohorts.

The deposition of lectin pathway elements has been associated with poorer outcomes in IgAN in several studies. MBL deposits, found in about 25–35% of patients, have been associated with higher proteinuria, lower eGFR, and more severe histopathological lesions (70, 75). Several retrospective studies have confirmed the deleterious prognostic impact of mesangial C4d deposition on renal survival (71, 76, 77). In those series, the prevalence of C4d positivity ranged from 21% in the pediatric cohort up to 38% in the adult Spanish cohort (71, 76, 77).

Mesangial co-deposition of complement elements highlights the pathophysiological role of activation of the alternative and lectin pathways in IgAN and can be considered to be a biomarker of the disease itself as well as its severity. The complexity of the combinations of those deposited proteins offers a potential approach to personalize complement-targeting therapies for patients in the future.

## Complement Fragments in the Circulation of IgAN Patients

Despite the presence of normal or elevated C3 levels in the circulation of most Caucasian patients with IgAN, C3 activation fragments are present in about 50% of patients (78). Subsequently, larger studies showed that 45% of the patients with IgAN had a significantly elevated C3dg level (79) and 70% of pediatric IgAN patients had significantly elevated C3d/C3 ratio in the circulation (80).

Two groups examined plasma levels of activated C3 (actC3) using somewhat similar monoclonal antibodies that detected neoantigens expressed after activation of C3 (81, 82). These monoclonal antibodies were produced in the laboratories of highly accomplished complement investigators, Drs. Eberhard (Scripps) and Götze (Göttingen). The neoantigen recognized by the Scripps antibody is on iC3b, C3dg, and C3d (83), while the Göttingen antibody recognized C3b, iC3b, C3dg, and C3d (81). Data generated from use of the Scripps antibody are available only for subjects with systemic lupus erythematosus (SLE) (84). Neither antibody appears to be available today.

ActC3 in plasma was detected on one occasion for 73% of 55 adult and 57% of 28 pediatric German patients with IgAN when compared to healthy controls (82). When compared to patients with non-immune renal diseases, an elevated plasma actC3 level was found in 30% of patients with IgAN (82). There was an association with progressive loss of renal function with a single elevated actC3 level, with 75% sensitivity and 89% specificity for predicting progression. Weak, but significant, correlations were shown for degree of proteinuria and microscopic hematuria. In a subsequent study of an expanded cohort, mean plasma C3a level was higher for patients with IgAN compared to healthy controls, but mean levels for patients with stable renal function or progressive disease were similar (85). Plasma actC3 levels were near normal for the US patients with normal or minimal mesangial changes, likely corresponding to an Oxford score of M0, E0, S0, T0, and C0 (81). Patients with mesangial proliferation, crescents, or segmental glomerulosclerosis had elevated levels compared to healthy adult controls (81). Plasma C3a levels were significantly elevated for 35% of 46 adult patients with IgAN or IgA vasculitis with nephritis (86). In this study, the plasma C3a level was significantly associated with serum creatinine concentration but not 24-h urinary protein excretion. In the expanded German cohort cited above, the mean plasma C3a level was higher in adult patients with IgAN as compared to healthy controls (85). In this study, plasma C3a level did not correlate with the plasma actC3 level and the mean level did not differ between patients with stable renal function or progressive dysfunction. These findings suggest that the plasma C3a level does not supplant the plasma actC3 level for predicting decline in renal function.

Prior to the delineation of the MBL pathway (87, 88), fragments generated by activation of C4 were considered evidence of activation of the classical pathway. As noted above, we now understand that, for patients with IgAN, they are more likely generated through the MBL pathway. Significant elevation of plasma C4d/C4 ratio was found on at least one occasion for 28% of adult and 11% of pediatric patients with IgAN (80). C4-C3 complexes, assumed to indicate activation of the classical pathway, were elevated in only 8% of patients with IgAN (82).

In two early studies, soluble C5b-9 levels were normal for pediatric and adult patients with IgAN (81, 82). However, another study reported significantly elevated plasma C5b-9 levels for 17% of adult patients with IgAN (79).

Serum complement levels have also been investigated as diagnostic tools. In a Japanese study including 418 healthy individuals, and 195 IgAN and 111 non-IgAN glomerular disease patients, the pre-biopsy ratio of serum IgA to C3 (IgA/C3) was highest among IgAN patients. Additionally, it was a good diagnostic marker to distinguish IgAN from other glomerular diseases. The higher serum IgA/C3 ratio in IgAN patients compared to that in non-IgAN glomerular disease patients was driven by not only a significantly lower C3 level but also a significantly higher IgA level (89). Several studies from East Asia also suggest that the IgA/C3 ratio can be used as a prognostic marker, with higher values being associated with more severe disease histology (90) and worse clinical outcomes including urinary protein excretion, hematuria, and higher creatinine level (91). Among Japanese patients treated with corticosteroids and tonsillectomy, a higher IgA/C3 ratio was associated with a higher incidence of disease recurrence (92).

### Complement Proteins and IgA-Containing Immune Complexes in IgAN

In IgAN, the presence of IgA-containing circulating immune complexes (93–97) and association of complement-containing immune complexes with disease activity have been observed in early studies, (98–101) with many of these observations clarified in follow-up studies, as detailed in the section above. It is now thought that the pathogenic IgA1-containing immune complexes, that can activate primary human mesangial cells in culture to proliferate and produce cytokines and extracellular matrix, play a key role in the pathogenesis of IgAN (19, 33, 96, 102–109). Moreover, studies of various animal models of IgAN also indicate complement involvement in disease development (14, 110).

Analysis of a model of immune complexes, heat-aggregated mixture of human IgG and IgA1, indicated that these mixed-immunoglobulin aggregates, but not IgA alone, activated C3 (111). Moreover, a study of IgA immune complexes formed *in vitro* from Gd-IgA1 and anti-glycan IgG antibodies in cord-blood serum indicated that the capacity of these complexes to activate proliferation of mesangial cells was dependent on a heat-sensitive serum factor, presumably complement (112). This model of formation of immune complexes *in vitro* was later enhanced by using recombinant Gd-IgA1-specific IgG derived from an IgAN patient (7, 112). Notably, these immune complexes, when formed in the presence of serum, also activate cultured primary human mesangial cells (102, 104, 105, 113).

C3 is present in IgA1-containing circulating immune complexes of patients with IgAN (114). A pilot study of IgA1-containing circulating immune complexes from IgAN patients as well as those formed *in vitro* indicated the presence of C3 products (115). Specifically, C3 α and β chains were detected in the active, large-molecular-mass immune complexes consisting of galactose-deficient IgA1 and recombinant IgG autoantibody. Targeted mass spectrometric analysis identified iC3b, C3c, and C3dg fragments in these complexes. Together, these findings are suggestive of direct binding of C3 and activation of the alternative pathway in this *in vitro* model of IgAN immune complexes (14, 35).

## Genetic Studies on the Role of Complement Proteins in IgAN

Genetic influences in the development of IgAN were first implicated by a 1985 study of a familial form of this disease (15). Although more studies followed [e.g., (116, 117)], a better appreciation of the impact had to wait until technical advances in genomics enabled genome-wide association studies (GWAS). GWAS of IgAN then provided the initial insight into the genetic architecture of IgAN by identifying specific susceptibility loci across cohorts from Europe, North America and East Asia (118–125). Common genetic variants (including those affecting the alternative complement pathway) may in part explain the geographical differences in disease prevalence worldwide (126). Serum levels of the autoantigen, Gd-IgA1, represent a heritable trait (127, 128) and two loci encoding a specific glycosylation enzyme and its chaperone are linked to this phenotype based on two recent GWAS publications (129, 130). A reader interested in more details on GWAS studies and genetics of IgAN is referred to more specialized reviews [e.g., (126, 131–135)]. Here, we will briefly present genetic and genomic data related to the role of complement in the pathogenesis of IgAN.

Among the loci associated with IgAN that are related to complement are single-nucleotide polymorphisms (SNPs) on chromosome 1q32 (centered on reference SNP ID number [rs] rs6677604) and 16p11 (rs11574637 and rs7190997). The locus on chromosome 1q32 includes a cluster of genes (*CFHR 1-5*) that encode factor H-related proteins and rs6677604 is a surrogate marker of *CFHR1,3* gene deletion (*CFHR3,1*Δ). This allele is associated with a reduced risk of developing IgAN. As CFHR peptides are involved in the regulation of the alternative pathway, the absence of these CFHR peptides may lead to a more potent inhibition of complement system by FH. This postulate is supported by a recent study; it showed that *CFHR3,1*Δ (heterozygous or homozygous) was associated with a reduced level of glomerular immune deposits (IgA, IgG, and C3) (63). These findings correspond well with the early observations about the involvement of the complement system in IgAN (35, 99) and the current understating of complement's role in IgAN (34, 136). Moreover, several studies confirmed that genetic variants in CFH, CFHR3,1, and possibly CFHR5, can differentially affect complement activation and, thus, impact predisposition to IgAN (34, 37, 137). For example, serum CFH levels are negatively associated with mesangial C3 deposition (37). For CFHR5, 28 rare and 4 common variants in amino-acid sequence were identified in a Chinese cohort and the distribution of rare variants in patients with IgAN differed significantly from that in controls (137). Moreover, some of the rare variants were functional, as shown by the reduced or increased C3b binding by recombinant CFHR5 variant proteins compared to the wild-type protein.

The second IgAN GWAS locus related to complement is on chromosome 16p11 that contains *ITGAM* and *ITGAX* genes that encode integrins αM and αX, respectively. These integrins have roles in the formation of leukocyte-specific complement receptors 3 and 4 by combining with the integrin β2 chain. ITGAM gene product, also known as CD11b, is the α-chain of the αMβ2 integrin. This leukocyte-specific integrin regulates cell activation and adhesion of neutrophils and monocytes, enabling endothelium stimulation and phagocytosis of complement-coated particles. This locus is associated with several other autoimmune diseases, including SLE (138, 139). The SLE-associated variant is related to a reduced clearance of immune complexes (140). α-X chain protein associates with β2-chain to form another leukocyte-specific integrin with functions thought to be similar to those of ITGAM.

Together, recent GWAS have currently identified 22 IgAN-associated loci. Within these loci are genes encoding products involved in complement regulation and interaction with immune complexes that may account for these associations with IgAN. These findings have provided novel insights about possible mechanisms of disease. Follow-up genetic and biochemical studies are needed to delineate the precise roles of these complement-associated genes and their alleles.

## Complement System as Target for Future Therapies

Recent scientific advances have improved our understanding of the role of complement in the pathogenesis of IgAN. This information has led to identification of new potential therapeutic targets to halt or slow the disease course. So far, treatment with complement inhibitors is limited to a few published cases reporting the use of eculizumab, a humanized monoclonal antibody that inhibits cleavage of C5 by C5 convertase, as rescue therapy. The first report came from Sweden; a young 16-year-old white male with biopsy-proven crescentic IgAN had failed to respond to corticosteroids and mycophenolate but stabilized when treated with eculizumab, although the therapeutic effects were not sustained (141). Similarly, another 16-year-old male with crescentic IgAN who had failed treatment with corticosteroids, cyclophosphamide, and plasma exchange subsequently had transient improvement in renal function with eculizumab (142). Knowing the role of complement in IgAN pathophysiology, and encouraged by these anecdotal therapeutic results, Herzog et al. used eculizumab as a rescue therapy in a 28-year-old male with post-transplant recurrent crescentic IgAN. The attempt to salvage the allograft failed but therapy was instituted after the initiation of dialysis and hence may have been too late (143). In a series of elegant experiments, Zhang et al. showed that antagonists of the receptors for C3 and C5 prevented proliferation of cultured human mesangial cells stimulated by IgA and reduced up-regulation of IL-6 and monocyte chemoattractant protein 1 (MCP-1) (144). In an experimental model of IgAN, mice deficient for C3 and C5 receptors had less proteinuria, mesangial IgA deposition, mesangial matrix expansion and hypercellularity than normal mice, but serum creatinine and blood urea nitrogen levels were similar. These experiments suggest that perhaps inhibition of receptors for C3 and C5 may be promising therapeutic interventions in the future (144).

**Figure 3:**
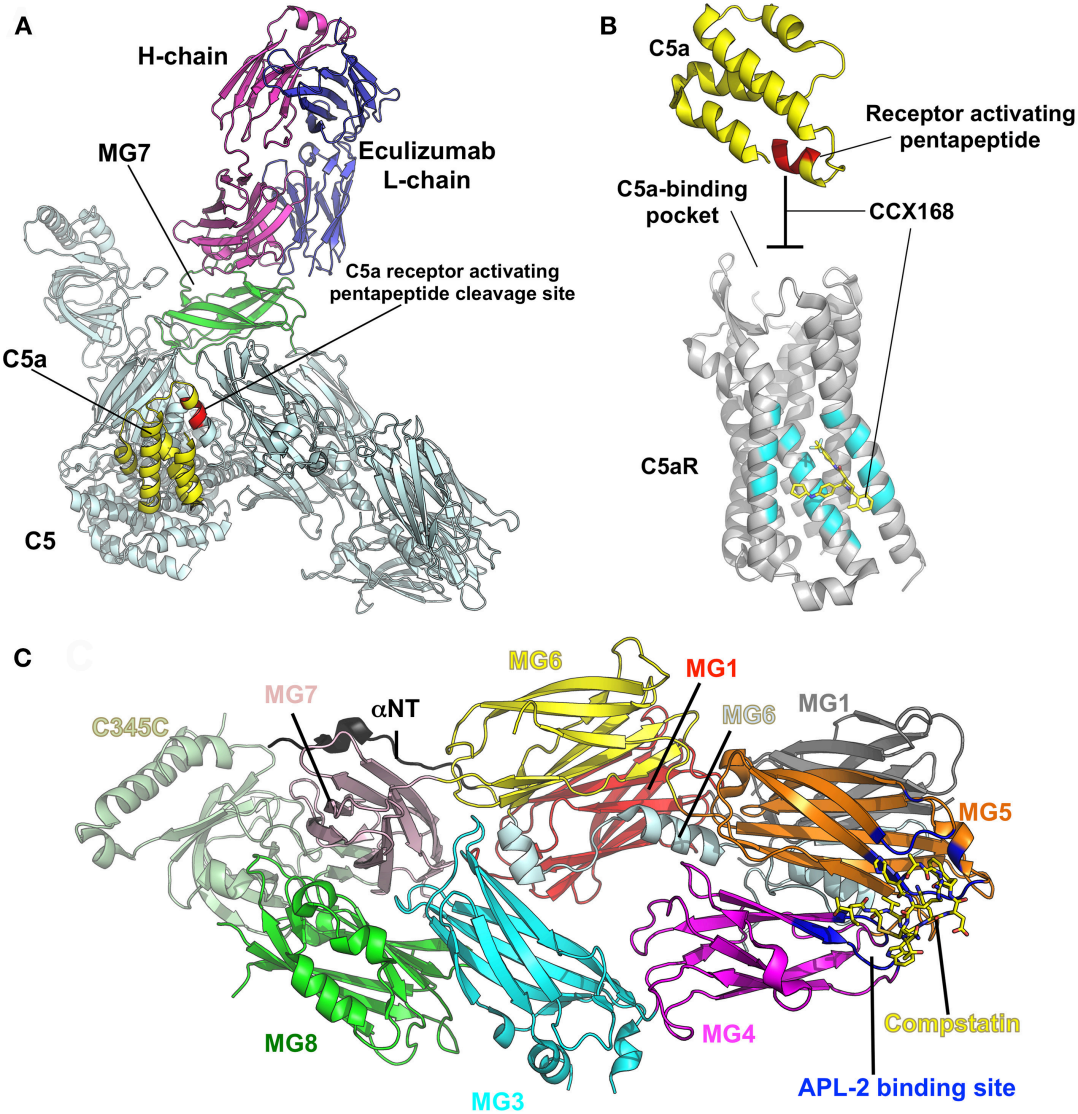
Examples of therapeutic control of complement-activation pathways. **(A)** The complex of C5 and the Fab derived from eculizumab antibody (PDB ID: 5I5K) (145) are shown in cartoon model with the heavy (H) and light (L) chains of the Fab colored in magenta and blue, respectively. C5 is shaded in pale cyan, while macroglobulin domain 7 (MG7), the site of eculizumab binding on C5, is highlighted in green. C5a is yellow and the C5a-receptor-activating pentapeptide cleavage site is red. **(B)** C5a (yellow) binds to the membrane-associated C5a receptor (C5aR, gray background) *via* the C-terminal C5a pentapeptide (red). CCX168 (Avacopan, yellow stick model) binds to the surface of C5aR (pocket shaded in cyan), thereby blocking C5a binding through allosteric effects on the C5a-binding pocket. The C5a/C5aR model is based on PDB ID: 6C1R (146). **(C)** Compstatin, and APL-2 are inhibitors of activation of C3. The crystal structure of C3c (PDB ID: 2QKI) (147), a major proteolytic fragment of C3, is shown in complex with compstatin (yellow stick model). Both inhibitors bind to a site (shaded in blue) formed by the macroglobulin domains 4 and 5 (MG4, MG5). All illustrations were prepared with PyMOL (148).

**Figure 4:**
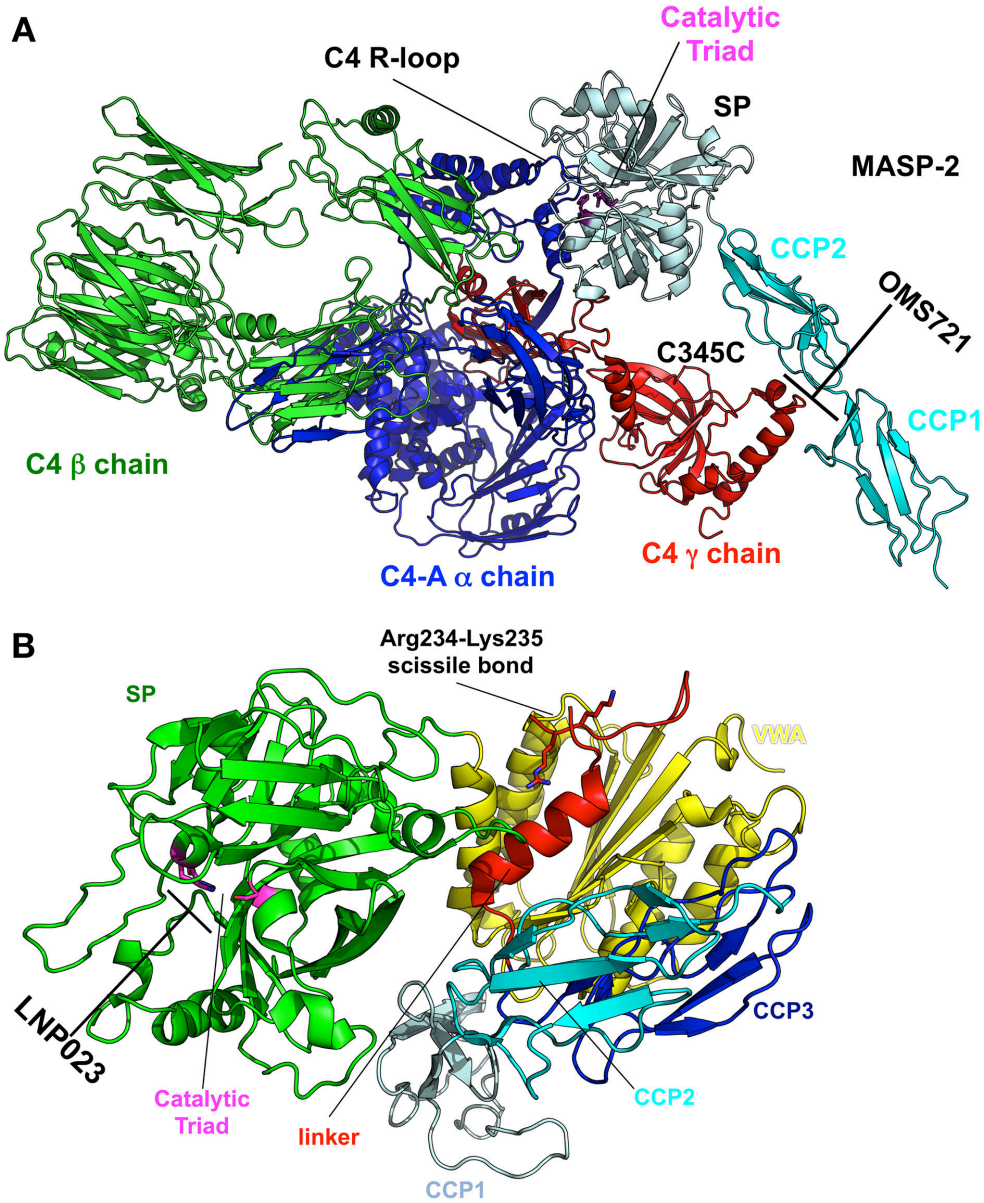
Targets and therapeutic agents of the lectin and alternative complement-activation pathways. **(A)** The crystal structure of the C4/MASP-2 complex is shown (PDB ID: 4FXG) (149). MASP-2 complement control protein (CCP) domains bind to C4. Upon association with MASP-2, C4 undergoes a conformational change whereby the scissile bond containing R-loop is inserted into the catalytic site of the serine-protease (SP) domain (pale cyan with catalytic triad in magenta). Cleavage yields fragments C4a and C4b. Monoclonal antibody OMS721 binds to a CCP domain of MASP-2, inhibiting the lectin pathway by inhibiting complex formation. **(B)** The structure of factor B is shown (PDB ID: 2OK5) (150). Upon C3b binding, the N-terminal CCP domains are displaced, leading to rearrangement of the central helices and release of the scissile bond for proteolytic activation. LNP023 inhibits the proteolytic activity of factor B.

Elucidating the role of activation of the MBL and alternative pathways in the pathophysiology of IgAN has identified new potential treatment targets. While some therapies (such as eculizumab and CCX168) may be non-specific inhibitors of the distal common pathway, others target a specific pathway more proximally (Figure 2). Inhibition of complement activation can be achieved with monoclonal antibodies, small molecules, and short peptides that block protein-complex formation and/or enzymatic activity. Eculizumab, a monoclonal humanized antibody, binds to complement protein C5 at the level of macroglobulin domain 7 (MG7), thus blocking cleavage of C5 by C5 convertase into pro-inflammatory components C5a and C5b (Figure 3A). C5a binds to the membrane-associated C5a receptor (C5aR) *via* the C-terminal C5a pentapeptide. CCX168 (Avacopan), a small molecule antagonist of the inflammatory response, binds to the surface of C5aR, thereby blocking C5a binding through allosteric effects on the C5a-binding pocket (Figure 3B). Compstatin, a cyclic tridecapeptide, and APL-2, a pegylated derivative of compstatin, inhibit the activation of C3 (Figure 3C). MASP-2 complement control protein (CCP) domain binds to C4. Upon association with MASP-2, C4 undergoes a conformational change whereby the scissile bond-containing R-loop is inserted into the catalytic site of the serine-protease domain. Cleavage yields fragments C4a and C4b. Monoclonal antibody OMS721 binds to a CCP domain of MASP-2, inhibiting the lectin pathway by blocking complex formation (Figure 4A). In the alternative pathway, FB binds to C3b displacing the N-terminal CCP domains. This in turn leads to rearrangement of the central helices and release of the scissile bond for proteolytic activation (Figure 4B). LNP023, an orally available small molecule, interferes with the alternative complement cascade by inhibition of the proteolytic activity of FB.

To date, clinical trials in the treatment of IgAN using surrogate end-points, such as doubling of serum creatinine, have been limited due to the variable course and often slowly progressing nature of the disease. The size and cost of such trials has been relatively prohibitive so far. More recently, and with guidance from the Unites States Food and Drug Administration, there has been renewed interest in testing novel therapies using “reasonably likely” surrogate end-points (such as quantitative proteinuria) that could lead to accelerated conditional drug approval (151). This change in policy has sparked the initiation of multiple clinical trials evaluating the benefits of various inhibitors of the complement cascade in IgAN (Table 1). Besides evaluating their efficacy, we need to assess the risks associated with the use of these drugs, infections being the primary concern. Very limited data regarding the safety of these inhibitors are available in the literature. In part, our understanding about the risks of drugs that interfere with the complement system comes from syndromes of congenital complement deficiencies. Observations that the infection rates in these children decreases as they age suggest that the role of the innate immunity becomes less prominent in the setting of maturing adaptive immunity (152). The risk of infection also depends on the level of pathway inhibition. While C5 inhibitors increase primarily the risk of neisserial infections, C3 inhibitors are likely to confer a broader infectious susceptibility warranting vaccination against several encapsulated organisms. However, even inhibitors like compstatin do not completely abrogate complement-mediated immunity against pathogens as even modest residual complement activity seems to be protective (55). Other theoretical safety concerns come from the observation that some classical complement deficiencies increase the risk of developing SLE, hence raising the concern for developing autoimmunity with complement inhibition (55). Perhaps the most information about drug safety comes from the use of eculizumab treatment that substantially increases the risk of infections with encapsulated organisms. In particular, the rate of meningococcal infection increases by 1,000-fold compared to that in the general population. It is recommended that patients contemplating treatment with eculizumab receive meningococcal vaccine at least 2 weeks prior to therapy initiation. Vaccination has reduced the risk of meningitis by 10-fold. If therapy is initiated prior to 2 weeks from the time of vaccination, antibacterial prophylaxis is recommended (153). Ultimately, the duration and extent of complement inhibition will also play a role in the safety of treatment. Several of these therapeutic agents are also being evaluated in a variety of other disorders ranging from atypical hemolytic uremic syndrome to age-related macular degeneration to various glomerulonephritides such as lupus nephritis and membranous nephropathy. The cumulative experience from all these trials will inform our future use of complement inhibitors in IgAN.

**Table 1:** Registered clinical trials of complement inhibitors being tested for the treatment of patients with IgAN.

**Drug**	**Type**	**Target**	**Trial**	**ID**	**Phase**	**Sponsor**
APL-2	Inhibitor	C3	Phase 2 study assessing safety and efficacy of APL-2 in glomerulopathies	NCT03453619	2	Apellis Pharmaceuticals LLC.
CCX168	Receptor antagonist	C5a receptor	Open-label study to evaluate safety and efficacy of CCX168 in subjects with IgAN on stable RAAS blockade	NCT02384317	2	ChemoCentryx
LNP023	Inhibitor	Factor B	Study of the safety and efficacy of LNP023 in patients with kidney disease caused by inflammation	NCT03373461	2	Novartis Pharmaceuticals
OMS721	mAb	MASP-2	Safety study of IgAN, lupus nephritis, membranous nephropathy and C3 glomerulopathy including dense deposit disease treated with OMS721	NCT02682407	2	Omeros Corporation
			Study of the safety and efficacy of OMS721 in patients with IgAN	NCT03608033	3	

Data from https://ClinicalTrials.gov, accessed on December 2, 2018. IgAN, IgA nephropathy;

RAAS, renin angiotensin aldosterone system;

*ID-ClinicalTrials.gov identifier*.

## Conclusion

In the past several decades, much progress has been made in understanding the role of the complement system in IgAN pathogenesis and prognosis. Data from studies of the pathology features, biochemistry of IgA1, and genetic influences on the disease and animal models confirm the involvement of the alternative and lectin pathways. Markers of complement activation are not only diagnostic but are also emerging as prognostic tools to risk-stratify disease severity. Complement components likely play significant roles in amplifying the inflammatory response for formation of immune complexes and their deposition in the glomerular mesangium. These findings have sparked marked interest in targeting the complement cascade at multiple levels in an effort to halt or slow the disease progression. Much remains to be learned about the optimal timing and intensity of use of complement inhibitors and their efficacy and safety in the treatment of patients with IgAN.

## Author Contributions

DR, JN, BJ, and RW conceived the general outline. BK and TG generated the figures, with feedback from JN, DR, and BJ. All authors (DR, NM, BJ, BK, TG, JN, RW) contributed intellectually by writing assigned sections, editing and revising the drafts, and proofreading the manuscript.

### Conflict of Interest Statement

DR, BJ, JN are co-founders of Reliant Glycosciences, LLC. The company did not have any role in the drafting of this manuscript. RW has consultation agreements with Omeros Corporation, Apellis Pharmaceuticals, Aduro Biotech, and Catabasis Pharmaceuticals. The remaining authors declare that the research was conducted in the absence of any commercial or financial relationships that could be construed as a potential conflict of interest.
